# siRNA-Mediated Reduction of Apolipoprotein CIII Delays Pancreatic Islet Deterioration and Onset of Type 1 Diabetes in Diabetes-Prone BioBreeding Rats

**DOI:** 10.3390/biomedicines14071481

**Published:** 2026-06-30

**Authors:** Patricia Recio-López, Pere Rehues, Per-Olof Berggren, Lisa Juntti-Berggren, Ismael Valladolid-Acebes

**Affiliations:** The Rolf Luft Research Center for Diabetes and Endocrinology, Karolinska Institutet, Karolinska University Hospital, Anna Steckséns Gata 53, SE-171 76 Stockholm, Sweden

**Keywords:** type 1 diabetes (T1D), apolipoprotein CIII (apoCIII), siRNA, islet transplantation

## Abstract

**Background/Objectives**: Type 1 diabetes (T1D) is an autoimmune disease characterized by progressive β-cell loss. Apolipoprotein CIII (apoCIII), a lipid metabolism regulator, is elevated in T1D and implicated in β-cell apoptosis. Antisense oligonucleotide–mediated apoCIII reduction delays diabetes onset in diabetes-prone BioBreeding (DPBB) rats. This study examined whether small-interfering RNA (siRNA) targeting apoCIII during the final prediabetic month preserves islet integrity and delays T1D onset. **Methods**: Two siRNAs targeting rat apoCIII were evaluated in 30-day-old DPBB rats for efficacy and off-target effects. Hepatic and plasma apoCIII levels were measured, and neighboring apolipoprotein gene expression was assessed. The most specific candidate (apoCIII-siRNA2) was selected. Duration of action was determined after a single injection. To study the effects of apoCIII-lowering treatment in vivo, islets from 25-day-old DPBB rats were transplanted into the anterior chamber of the eye of age-matched DPBB recipients. Rats received weekly intravenous injections of apoCIII-siRNA2 from day 30 until diabetes onset. Islet morphology, vascularization, and phagocyte infiltration were assessed by confocal imaging three and five weeks post-transplantation. **Results**: Both siRNAs reduced apoCIII, but one showed off-target effects and was excluded. A single injection of apoCIII-siRNA2 suppressed plasma apoCIII for approximately one week and weekly treatment maintained low circulating apoCIII levels. Five weeks after transplantation islet morphology and vascularization were preserved, and there was no increase in phagocyte infiltration. This resulted in a delayed onset of diabetes. **Conclusions**: siRNA-mediated apoCIII reduction delays pancreatic islet deterioration and T1D onset in DPBB rats, supporting apoCIII as a contributing factor to β-cell vulnerability and thereby a potential therapeutic target.

## 1. Introduction

Type 1 diabetes (T1D) is an autoimmune disease characterized by progressive destruction of pancreatic β-cells. Over recent decades, there has been an increase in the incidence of T1D, accompanied by a shift toward younger age at diagnosis [1,2]. T1D is believed to arise from a complex interplay of genetic predisposition, immune dysregulation, and environmental influences. Despite intensive research, the initiating and driving factors underlying T1D remain largely unidentified. The relative contribution of these factors likely differs between individuals.

Apolipoprotein CIII (apoCIII) is a small protein predominantly synthesized in the liver, but we have previously demonstrated its expression also in pancreatic islets [3]. Exposure of β-cells to serum from newly diagnosed T1D patients induces Ca^2+^-mediated apoptosis, with apoCIII identified as the responsible factor [4,5]. Subsequent studies demonstrated that increased levels of apoCIII hyperactivates β-cell Ca_V_1 channels through SR-BI/β1 integrin-dependent coactivation of PKA and Src kinase signaling pathways [6].

The diabetes-prone BioBreeding (DPBB) rat spontaneously develops T1D around postnatal day 60, with both males and females developing the disease [7,8,9]. Similar to human T1D, disease onset is preceded by insulitis [10,11,12]. ApoCIII is upregulated in islets from DPBB rats compared with islets from diabetes-resistant BB (DRBB) rats [13]. This upregulation is accompanied by increased expression of markers of phagocyte and immune-cell infiltration (*Iba1*, *Mcp1*, and *Cd4*), elevated *Tnf-α* expression, and activation of inflammasome-related genes, resulting in increased *Il1-β* levels. Immunostaining of pancreatic sections further confirmed elevated IL-1β levels in DPBB rat islets compared with DRBB rat islets. Reduction of endogenous apoCIII levels in DPBB rats between days 12 and 40, using antisense oligonucleotides (ASOs), significantly delayed onset of disease [13].

In addition to its established role in lipid metabolism [14,15,16,17], elevated apoCIII levels in normolipidemic individuals with T1D have been associated with increased risk of cardiovascular disease [18]. Moreover, higher apoCIII levels independently correlate with microvascular complications in patients with T1D [19]. Certain apoCIII gene haplotypes associated with increased protein expression have been linked to a higher risk of developing T1D [20].

As small-interfering RNA (siRNA)-mediated silencing has shown greater potency and durability than ASO-based approaches [21], in the present study we investigated whether reducing apoCIII using siRNA during the last period of the prediabetic phase could preserve islet integrity and influence the onset of diabetes. Our working hypothesis was that siRNA could be administered at a lower dose and less frequently while achieving an effect on delaying diabetes onset that was comparable to or better than that of ASO.

We utilized the technique developed in our laboratory in which pancreatic islets are transplanted into the anterior chamber of the eye (ACE), enabling longitudinal, non-invasive in vivo imaging and assessment of islet morphology and viability [22].

## 2. Materials and Methods

### 2.1. Animals

BioBreeding (BB) rats were obtained from our breeding colony at Karolinska Institutet. The incidence of diabetes among our diabetes-prone (DP) BB rats is 100% in both males and females, and the average age of onset is 60 days. Diabetes onset is defined as blood glucose levels ≥ 15 mmol/L for three consecutive days. Diabetes-resistant (DR) BB rats do not develop diabetes.

The animals were housed under specific pathogen-free conditions in a temperature- and humidity-controlled room with 12 h light:dark cycles. They were fed the R70 chow diet (Lantmännen, Stockholm, Sweden) and water ad libitum. All animal care and experiments were carried out according to the Animal Experiment Ethics Committee at Karolinska Institutet. Rats were 25 days old at the start of the experiments, unless otherwise specified. Both male and female rats were included.

### 2.2. Isolation of Islets and Transplantation into the Anterior Chamber of the Eye (ACE)

Donor rats were euthanized using CO_2_, and the pancreas was perfused with 1 mg/mL collagenase A (Roche) diluted in Hanks’ Balanced Salt Solution (HBSS; ThermoFisher Scientific, Waltham, MA, USA) supplemented with 0.5% (*w*/*v*) bovine serum albumin (BSA; Sigma-Aldrich, St. Louis, MO, USA) and 25 mM HEPES (ThermoFisher Scientific), buffered to pH 7.4 (HBSS-BSA buffer). After pancreatic distension, a total pancreatectomy was performed, and the pancreas was digested at 37 °C in a water bath with continuous gentle shaking for 13 min. Digestion was terminated by adding ice-cold HBSS-BSA buffer. Islets were isolated from the exocrine tissue by handpicking under a stereomicroscope. Isolated islets were cultured in RPMI 1640 medium (ThermoFisher Scientific) supplemented with 10% (*v*/*v*) fetal bovine serum (FBS; Gibco, Waltham, MA, USA), 2 mM L-glutamine (Gibco), and penicillin–streptomycin (100 U/mL penicillin and 100 μg/mL streptomycin; Gibco). Islets were maintained at 37 °C in a humidified atmosphere of 5% CO_2_ and 95% air for 24 h prior to transplantation.

Recipient rats were anesthetized with isoflurane (2.5–3%) delivered in oxygen (400–500 mL/min). Islets were transplanted into the ACE as previously described [22], following the protocol outlined in Figure S1A.

### 2.3. In Vivo Imaging of Transplanted Islets and Image Analysis

Transplanted islets were imaged in vivo as previously described [22] using a TCS SP5 II laser scanning confocal microscope (Leica Microsystems, Wetzlar, Germany) equipped with water-dipping objectives (Leica HCX IRAPO L 25.0 × 0.95 and HXC-APO10x/0.30 NA). Viscotears (Bausch + Lomb) was used as the immersion liquid between the objective and the eye. Backscatter reflected light images were obtained using a 633 nm laser beam. To evaluate vascularization, a tetramethylrhodamine (TMR)-conjugated dextran, 2000 kDa (ThermoFisher Scientific), was injected intravenously (i.v.) at a dose of 0.5 mg per rat. Blood vessels were imaged directly after injection, while phagocytes were imaged 24 h later.

Volumetric quantification of vessel and phagocyte density was performed using Volocity Software (version 6.0.1; PerkinElmer, Waltham, MA, USA). Densities were calculated by normalizing vessel and phagocyte volume to the islet volume.

### 2.4. siRNAs

Two commercially available, active double-stranded siRNAs targeting the rat *apoCIII* gene, apoCIII-siRNA1 and apoCIII-siRNA2 (ThermoFisher Scientific), were used. The sequences of the active siRNAs are provided in Table S1. A non-targeting control siRNA (Silencer™ Select Negative Control siRNA, ThermoFisher Scientific) was used as a negative control. All siRNAs (active and control) were formulated using Invivofectamine™ 3.0 Reagent (ThermoFisher Scientific).

To test siRNA efficacy, 30-day-old DPBB rats were divided into four treatment groups. Three groups received a single i.v. injection of apoCIII-siRNA1, apoCIII-siRNA2, or control siRNA, respectively, at a dose of 1 mg/kg. The fourth group received an equivalent volume of phosphate-buffered saline (PBS, vehicle). Rats were euthanized 24 h post-injection, and liver, small intestine, and blood (for plasma collection) were harvested for apoCIII determination. Liver and intestinal tissues were snap-frozen in liquid nitrogen. Blood samples were centrifuged at 10,000× *g* for 15 min at 4 °C, and the plasma-containing supernatant was collected. Plasma and tissue samples were stored at −80 °C until analysis.

Off-target effects of the siRNAs were assessed by measuring the gene expression of apolipoproteins AI, AIV, and AV.

### 2.5. Duration of the Gene Silencing Effect by the siRNA

To evaluate the duration of the apoCIII-lowering effect of the selected siRNA, 30-day-old DPBB rats received a single i.v. injection of apoCIII-siRNA2 (1 mg/kg). Blood samples for plasma apoCIII analysis were collected before injection (time point 0) and at multiple time points thereafter, with the final sample collected 31 days post-injection.

### 2.6. Treatment with apoCIII-siRNA2

Islets were isolated from 25-day-old DPBB rats and transplanted into age-matched DPBB recipients. Transplanted animals received weekly i.v. injections of apoCIII-siRNA2 (1 mg/kg), starting at 30 days of age and continuing until the onset of T1D (Figure S1A).

Transplanted islets were imaged for vascularization and phagocyte infiltration three and five weeks after transplantation, as previously described [22].

### 2.7. RNA Isolation, cDNA Synthesis, and Quantitative Real-Time PCR (qRT-PCR)

Total RNA was isolated from freshly frozen tissues using the RNeasy Lipid Tissue Mini Kit (Qiagen, Venlo, The Netherlands), following the manufacturer’s instructions. RNA concentrations were determined using a NanoPhotometer P330 (IMPLEN, Munich, Germany). A total of 500 ng of RNA was used for cDNA synthesis with the High-Capacity cDNA Reverse Transcription Kit (Applied Biosystems, Waltham, MA, USA), according to the manufacturer’s instructions. qRT-PCR was performed using a QuantStudio 5 thermal cycler (Applied Biosystems) with PowerUp SYBR Green PCR Master Mix (Applied Biosystems). Gene expression analysis was carried out using the ΔΔCt method. Transcript levels of target genes were normalized to the cycle threshold (Ct) value of a reference gene specific to each tissue. As control genes, glyceraldehyde-3-phosphate dehydrogenase (*GAPDH*) was used for the liver; *GAPDH*, TATA-binding protein (*TBP*), and ribosomal protein S29 (*RPS29*) for the intestine; and *TBP* for the islets. Results are expressed as mRNA levels relative to vehicle-treated and/or control groups. Primer sequences are provided in Table S2.

### 2.8. Western Blot

ApoCIII was determined in plasma. Protein concentrations were measured by Bradford assay (BioRad, Hercules, CA, USA) and equal amounts of protein (50 µg) were loaded onto a 4–12% Bis-Tris electrophoresis gels (Invitrogen, Carlsbad, CA, USA).

For immunoblotting, membranes were blocked with 5% (*w*/*v*) BSA, in 0.1% (*v*/*v*) Tween-20 (Sigma-Aldrich) in PBS (PBS-T). After blocking, membranes were probed with primary antibodies overnight at 4 °C, then washed in PBS-T with 0.05% (*w*/*v*) BSA and incubated with the secondary antibodies for one hour at room-temperature. Thereafter, membranes were exposed to commercially enhanced chemiluminescence reagents (ClarityTM Western ECL Substrate, BioRad), and blots developed in a luminescent image analyzer (ChemiDocTM Touch Imaging System, BioRad). Transferrin was used as loading control. Densitometry analyses were performed in the obtained images using ImageJ software (version 1.54p, NIH, Bethesda, MD, USA).

The antibodies are listed in Table S3.

### 2.9. Statistical Analyses

Statistical analyses were performed using GraphPad Prism 5.0. Data are presented as mean ± SEM. One-way analysis of variance (ANOVA) followed by Tukey’s post hoc test was used for comparisons among three or more groups. Repeated-measures ANOVA followed by Dunnett’s post hoc test was used to evaluate longitudinal differences at various time points relative to baseline. For paired comparisons between two time points, a two-tailed paired Wilcoxon signed-rank test was used. Statistical significance was defined as *p* < 0.05.

## 3. Results

### 3.1. Evaluation of Two apoCIII Lowering siRNAs

**Figure 1:**
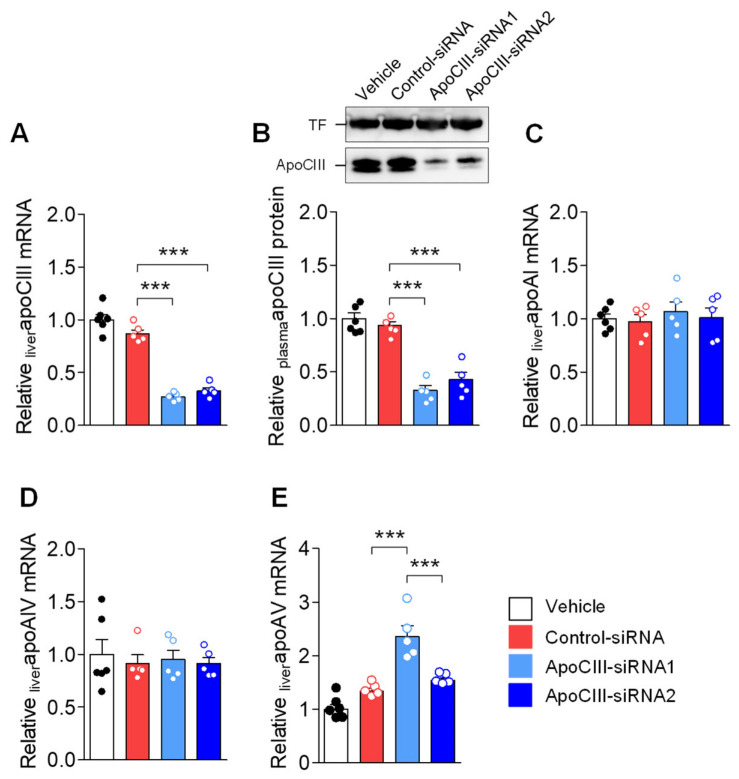
Evaluation of siRNA-mediated effects and off-target responses in liver and plasma. (**A**) Liver mRNA levels of *apoCIII*. (**B**) Representative immunoblots and densitometry analysis of apoCIII in plasma. mRNA levels of (**C**) *apoAI*, (**D**) *apoAIV* and (**E**) *apoAV* in liver. *N* = 5–6. Data are expressed as mean ± SEM. One-way-ANOVA followed by Tukey’s post-hoc test were used. *** *p* < 0.001.

When reducing apoCIII with ASO, during a period of the prediabetic phase, the delay in T1D onset in DPBB rats was comparable to that in several humans [9]. Another method to target specific genes is the siRNA technology, and there are already drugs in clinical use based on this technology [23,24,25,26]. We have developed and used siRNAs against apoCIII in a mouse model for the metabolic syndrome [21]. Here we tested two commercially available siRNAs against rat apoCIII. Both siRNAs reduced the expression of liver and circulating plasma levels of the apolipoprotein, and inactive siRNA did not have any effect (Figure 1A,B).

**Figure 2:**
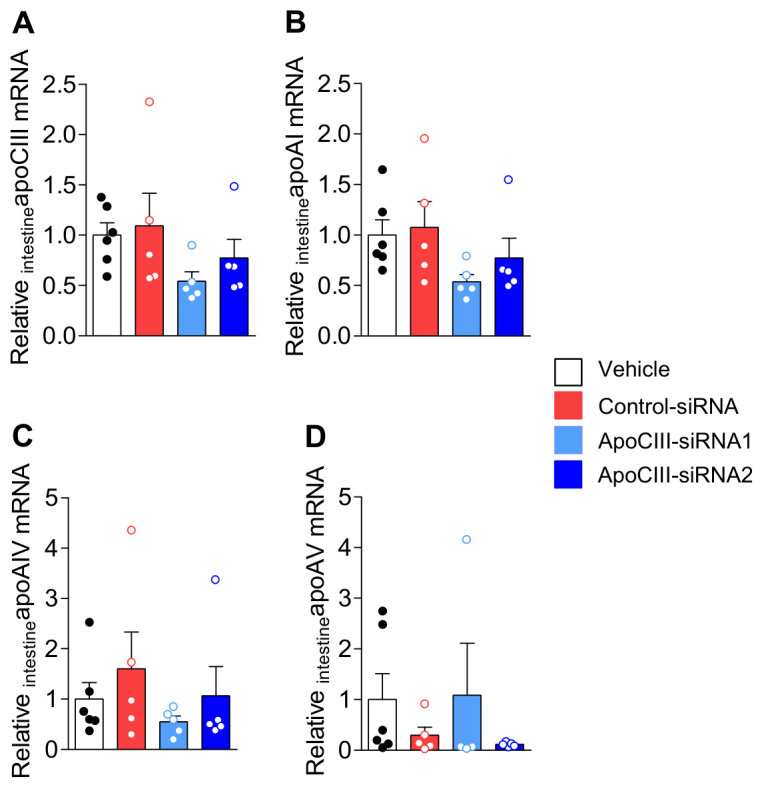
Effect of rat apoCIII-siRNAs on intestinal expression of apolipoproteins in the apoCIII gene cluster. Intestine mRNA levels of (**A**) *apoCIII*, (**B**) *apoAI*, (**C**) *apoAIV*, and (**D**) *apoAV* Data are expressed as mean ± SEM in *N* = 5–6. One-way-ANOVA followed by Tukey’s post-hoc test was used.

As the apoCIII gene is located in a cluster together with apolipoprotein AI, AIV, and AV, off-target effects of the siRNAs were evaluated (Figure 1C–E). In the liver, apoCIII-siRNA1, besides decreasing apoCIII, increased *apoAV*, while apoCIII-siRNA2 only affected the expression of apoCIII and therefore was chosen in our study (Figure 1E). None of the siRNAs affected intestinal expression of these genes (Figure 2A–D).

**Figure 3:**
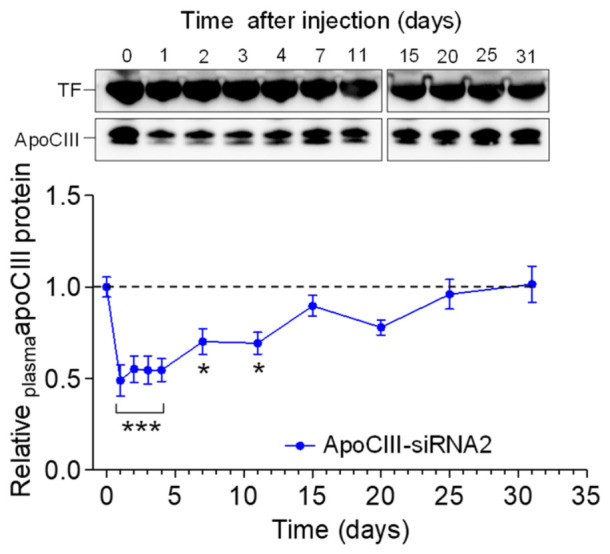
Duration of effect after a single injection of rat apoCIII-siRNA2. Duration of the apoCIII lowering effect of a single dose of the apoCIII-siRNA2 was evaluated by analyzing samples before and at the indicated time points after the injection. ApoCIII levels were normalized to baseline. *N* = 5. Data are expressed as mean ± SEM. Repeated-measures ANOVA followed by Dunnett’s post-hoc test was used. * *p* < 0.05; *** *p* < 0.001.

After a single injection of apoCIII-siRNA2, there was a fast decrease in the plasma levels of apoCIII that lasted for about one week, followed by a progressive increase reaching the basal level after 25 days (Figure 3). Based on these results, we decided to treat the animals once a week for a longer period of time.

### 3.2. Treatment with siRNA

Islets from 25 days old DP rats were isolated and transplanted into the ACE of DP rats of the same age. The recipients were treated from 30 days of age, once per week, with apoCIII-siRNA2 until T1D onset (Figure S1A).

**Figure 4:**
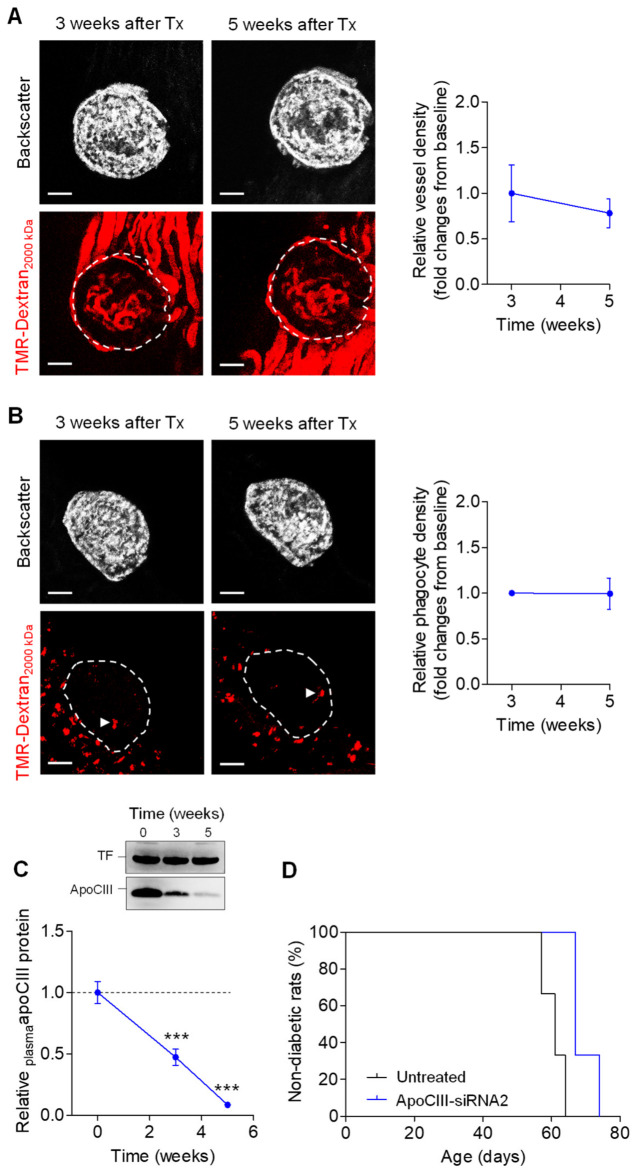
Lowering apoCIII preserves the islet morphology and vascular network in DP islets in DP recipients. (**A**,**B**) In vivo images of islets in the ACE of DP_islet_ → DP_rat,_ treated with apoCIII-siRNA2, three and five weeks after transplantation. (**A**) Functional vessels were analyzed after injection of TMR-dextran_2000 kDa_ and quantitative analysis of vessel density is shown in the graph next to the microphotographs. (**B**) Intra-islet infiltration of phagocytes, indicated with white triangles, are shown 24 h after the injection of TMR-dextran_2000 kDa_. Quantitative analysis of phagocytes is shown in the graph next to the in vivo images. In (**A**,**B**), dashed circles denote the islets. Scale bars are 50 µm. (**C**) Representative immunoblots and densitometry analysis of apoCIII in plasma during ongoing weekly treatment with apoCIII-siRNA2. The dashed line in (**C**) represents the pre-treatment baseline. (**D**) Effect of apoCIII-siRNA2 on T1D onset. *N* = 3. Data are expressed as mean ± SEM. In the quantitative analyses for panels (**A**,**B**), the two-tailed paired Wilcoxon signed-rank test was used. In panel (**C**) Repeated-measures ANOVA followed by Dunnett’s post-hoc test was used. *** *p* < 0.001.

The islets were imaged three and five weeks after transplantation, and morphology, vascularization, and presence of phagocytes were unaffected (Figure 4A,B). There was a normal age-related increase in BW, and the animals were normoglycemic at the final imaging session. (Figure S1B,C). Plasma apoCIII protein levels were reduced (Figure 4C) and the siRNA treatment delayed, but did not prevent, the onset of T1D (Figure 4D).

## 4. Discussion

Sera from T1D patients contain increased levels of apoCIII, as insulin deficiency upregulates the expression of this apolipoprotein [27]. Exposure of β-cells to T1D serum induces apoptosis [4,5]. ApoCIII is expressed in pancreatic islets and increases in parallel with the development of insulin resistance [3]. We have also shown that apoCIII levels are elevated in islets from prediabetic BB rats [13]. Furthermore, lowering apoCIII using ASOs prolonged the time to diabetes onset in diabetes-prone (DP) rats [9].

In a mouse model of metabolic syndrome, we previously used both ASOs and siRNAs to reduce apoCIII levels [13,17]. As siRNA-mediated silencing proved to be more potent and longer-lasting, we aimed to evaluate this approach in our T1D animal model. The onset of diabetes can vary between different strains of BB rats. In our breeding colony, however, diabetes develops within a narrow time frame of approximately 60 days, which is advantageous for intervention studies. Several studies have characterized insulitis and β-cell mass in DPBB rats and, as in humans, the onset of T1D is preceded by insulitis [10,11,12].

Like many other organs, β-cells have a substantial functional reserve, and overt T1D develops only after a major loss of β-cell function. This contrasts with T2D, in which hyperglycemia arises from multiple factors, including β-cell dysfunction, obesity, and insulin resistance. Consequently, disease onset is typically more gradual and occurs over a longer period than in T1D. In studies of T2D, metabolic evaluations often include glucose and insulin tolerance tests and measurements of C-peptide, which are important both for diagnosis and for assessing treatment effects. In T1D, persistent hyperglycemia reflects an absolute deficiency of insulin, and insulin replacement therapy is required for survival.

Because our mouse-specific siRNAs were ineffective in rats, we used commercially available rat-specific siRNAs targeting apoCIII. Two candidates were tested. Although both reduced apoCIII levels, one exhibited an off-target effect on apoAV and was therefore excluded.

In the present study, prediabetic islets were transplanted into the ACE of 25-day-old DP rats, which were then treated with apoCIII-siRNA2 once weekly starting at day 30. Five weeks after transplantation, the islets retained normal morphology and vascular structure and showed no signs of inflammation, despite being in a prediabetic environment.

However, although this treatment delayed onset of diabetes, it did not prevent it. The delay was less pronounced than that observed with ASO treatment [9] and corresponds to approximately one human year [28]. For comparison, teplizumab, a monoclonal anti-CD3 antibody, is approved for individuals ≥8 years of age with stage 2 type 1 diabetes to delay progression to stage 3 disease. The average time to progression to stage 3 was around 50 months in individuals treated with teplizumab compared with 25 months in those receiving a placebo [29].

In the ASO study, treatment was initiated earlier (at 12 days of age) and administered twice weekly until 40 days of age. In contrast, in the current study, treatment began later, approximately one month before the expected onset of diabetes. This timing may have been suboptimal, as the autoimmune process could already have progressed beyond a reversible stage, thereby limiting the impact of apoCIII reduction.

Another consideration is that the siRNA used in this rat study was less effective and had a shorter duration of action than the siRNAs used in our previous mouse study [21].

A limitation of this study is the absence of a control group treated with inactive siRNA. This decision was made in accordance with the 3Rs principle to reduce animal use and was supported by the evaluation of the two siRNAs, in which the inactive siRNA showed no effect (Figure 1A–E). In addition, historical control data from our ASO study showed no effect of scrambled ASO [9]. Furthermore, we have previously shown that healthy islets transplanted into the ACE of untreated 25-day-old DP rats become dysfunctional within five weeks after transplantation [13].

In conclusion, these findings, together with our previous work, support the potential of apoCIII-lowering therapies as a strategy to prevent or delay β-cell destruction in type 1 diabetes.

## Data Availability

The data that support the findings of this study are available from the corresponding authors upon reasonable request.

## Supplementary Materials

The following supporting information can be downloaded at: https://www.mdpi.com/article/10.3390/biomedicines14071481/s1, Figure S1. Experimental design of the treatment study with apoCIII-siRNA2 and evaluation of metabolic parameters. Table S1. Sequences of rat apoCIII-siRNAs. Table S2. Primer list for gene expression by qRT-PCR. Table S3. Antibody list for protein detection by Western blot.

## Abbreviations

The following abbreviations are used in the manuscript:

ApoCIII	Apolipoprotein CIII
ASO	Antisense Oligonucleotides
DRBB	Diabetes Resistant BioBreeding rats
DPBB	Diabetes Prone BioBreeding rats
T1D	Type 1 Diabetes
