# Supplementary material for: siRNA-Mediated Reduction of Apolipoprotein CIII Delays Pancreatic Islet Deterioration and Onset of Type 1 Diabetes in Diabetes-Prone BioBreeding Rats
Source: Biomedicines. 2026 Jun 30;14(7):1481. doi: 10.3390/biomedicines14071481 (PMC13405861; doi:10.3390/biomedicines14071481)
Supplement: Supplementary file 1 [file biomedicines-14-01481-s001.zip › Figure S1.pdf]

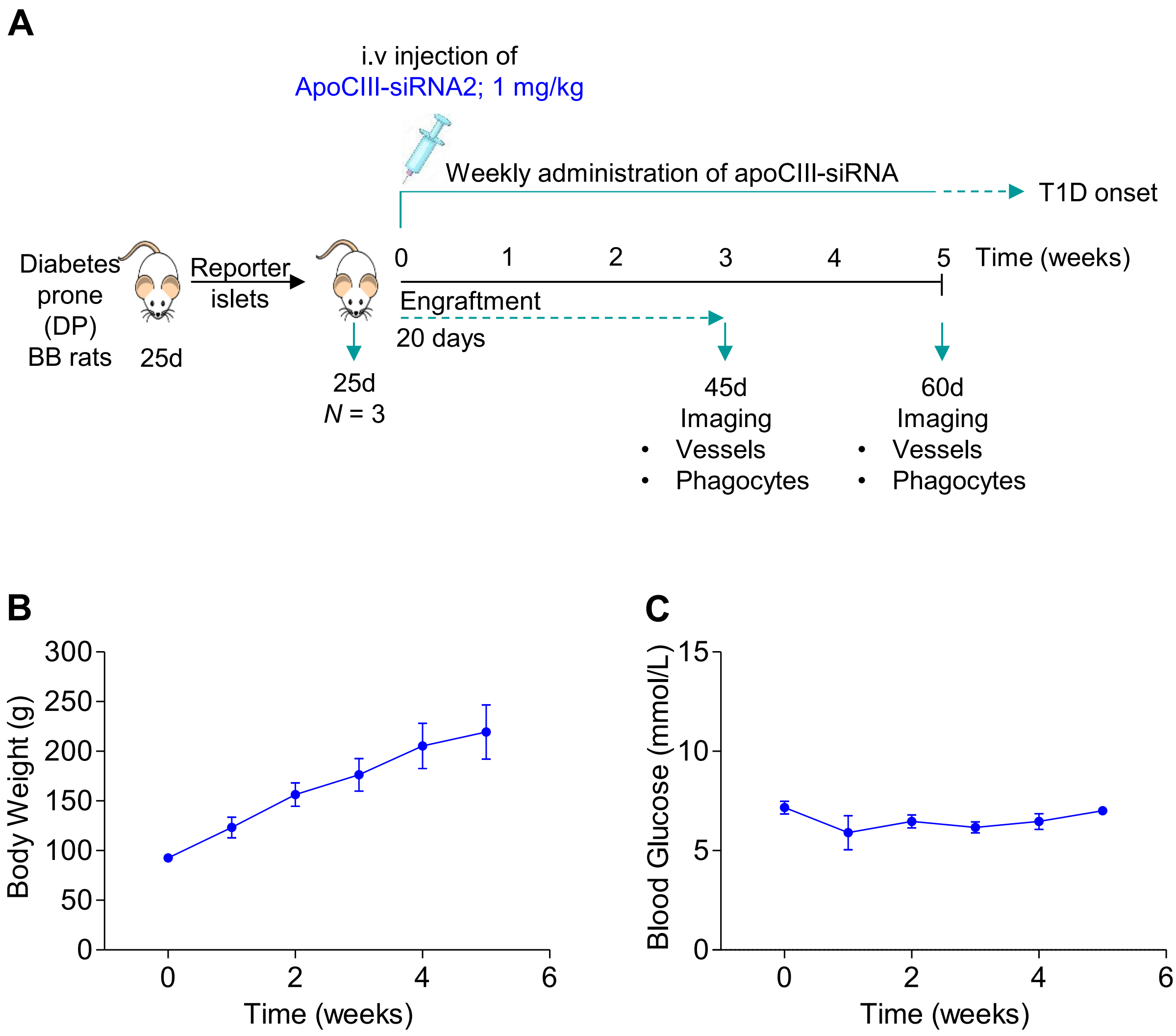

Figure S1: Experimental design of the treatment study with apoCIII-siRNA2 and evaluation of metabolic parameters. (**A**) Study protocol, (**B**) body weight and (**C**) non-fasting glucose. Data are presented as mean ± SEM of *N* = 3.
