# Supplementary material for: siRNA-Mediated Reduction of Apolipoprotein CIII Delays Pancreatic Islet Deterioration and Onset of Type 1 Diabetes in Diabetes-Prone BioBreeding Rats
Source: Biomedicines. 2026 Jun 30;14(7):1481. doi: 10.3390/biomedicines14071481 (PMC13405861; doi:10.3390/biomedicines14071481)
Supplement: Supplementary file 1 [file biomedicines-14-01481-s001.zip › Table S1.pdf]

Table S1. Sequences of rat apoCIII-siRNAs.

siRNA	siRNA ID	Sense sequence (5′→ 3′)	Antisense sequence (3′→ 5′)
**apoCIII-siRNA1**	s127462	GCAAGUUCACUGAUAAGUUTT	AACUUAUCAGUGAACUUGCTC
**apoCIII-siRNA2**	s127463	GAUGGACAAUCGCUUCAAATT	UUUGAAGCGAUUGUCCAUCCA
