# Supplementary material for: siRNA-Mediated Reduction of Apolipoprotein CIII Delays Pancreatic Islet Deterioration and Onset of Type 1 Diabetes in Diabetes-Prone BioBreeding Rats
Source: Biomedicines. 2026 Jun 30;14(7):1481. doi: 10.3390/biomedicines14071481 (PMC13405861; doi:10.3390/biomedicines14071481)
Supplement: Supplementary file 1 [file biomedicines-14-01481-s001.zip › Table S2.pdf]

Table S2. Primer list for gene expression by qRT-PCR.

**Gene ID**	**Primer strand**	**Primer sequence (5´→3´)**	**PCR size (bp)**
ApoAI	Forward	CAGGGTGAAGGATTTCGCCA	117
	Reverse	TCCAGGAGATTCAGGTTCAGC	
ApoAIV	Forward	TCACTCAACAGCTCAATACCCT	198
	Reverse	CTTTGTTGGCATGGGGCATC	
ApoAV	Forward	GTTCCAGGCTCGCTACTTTG	98
	Reverse	ACAGTTGCAAACACTGAGAGG	
ApoCIII	Forward	CCGAGCTGATGAGGGAGA	66
	Reverse	GAGGCTTGTTCCATGTAGCC	
GAPDH	Forward	CAACTCCCTCAAGATTGTCAGCAA	118
	Reverse	GGCATGGACTGTGGTCATG	
RPS29	Forward	CTCGTTCCTTTTTCCTCCTTGG	114
	Reverse	TTAGAGCAGACGCGGCAAG	
TBP	Forward	ACCGTACATCTCAGCTGCTTC	87
	Reverse	ATGAAACAGTGATGTGGGGACA	
