# Supplementary material for: siRNA-Mediated Reduction of Apolipoprotein CIII Delays Pancreatic Islet Deterioration and Onset of Type 1 Diabetes in Diabetes-Prone BioBreeding Rats
Source: Biomedicines. 2026 Jun 30;14(7):1481. doi: 10.3390/biomedicines14071481 (PMC13405861; doi:10.3390/biomedicines14071481)
Supplement: Supplementary file 1 [file biomedicines-14-01481-s001.zip › Table S3.pdf]

Table S3. Antibody list for protein detection by Western blot.

Protein	Molecular Weight	Primary ab	Primary ab dilution	Secondary ab	Secondary ab dilution
**ApoCIII**	8.8 kDa	Anti-ApoCIII (Rabbit; US Biological Life Sciences)	1:200	HRP-linked-anti-rabbit IgG	1:4000
**Transferrin**	80 kDa	Anti-TF (Rabbit; Thermo Fisher Scientific)	1:500	HRP-linked-anti-rabbit IgG	1:4000
